# When waters of the river and the well meet

**DOI:** 10.1111/1759-7714.13994

**Published:** 2021-05-10

**Authors:** Alan D. L. Sihoe

**Affiliations:** ^1^ Gleneagles Hong Kong Hospital Hong Kong China; ^2^ International Medical Centre Hong Kong China

There is a famous idiom in China: 河水不犯井水 (“the waters of the river and of the well do not mix”). This describes the state of two parties each going their own way without interfering with the other. It also aptly describes the traditional relationship between many surgeons and oncologists when managing lung cancer. The former treats patients with early‐stage disease, the latter deals with patients suffering advanced stage disease, and (almost) never the twain shall meet.

When the results of the PACIFIC trial were announced in 2017, this archaic divide seemed at first to further deepen.^1^ With modern immunotherapy, oncologists appeared to have acquired a powerful new weapon against lung cancer that suggested all other modalities of treatment might soon be rendered obsolete. The efficacy of durvalumab in prolonging survival in stage III non‐small cell lung cancer (NSCLC) patients after concurrent chemotherapy and radiotherapy appeared to be so great that the previous paradigm of following induction therapy with surgery was under threat. Prominent lung cancer physicians openly voiced their concerns that henceforth surgery might no longer have any role to play in stage III NSCLC.^2^ If oncologists moved onto this piece of turf, the surgeons would be forced to leave.

However, the dust soon settled and it was realized that the advent of immunotherapy may bring a new paradigm. Far from widening the chasm between specialties, it may bring them together. Early studies demonstrated the ability of immunotherapy to effect significant a major pathological response (MPR) in NSCLC, prompting the hypotheses that it would be a promising agent for neoadjuvant/induction therapy prior to surgery.^3^ Today, a number of phase III clinical studies are already well underway to explore the use of perioperative immunotherapy for NSCLC patients, including the CheckMate‐816 (NCT02998528), KEYNOTE‐671 (NCT03425643), IMpower‐030 (NCT03456063), and AEGEAN (NCT03800134) trials.^4^ Should the results of these trials indicate benefit for patients, the future of lung cancer management would witness closer co‐operation between oncologists and surgeons than ever before.

Should this come to pass, the merging of the river water and the well water will create turbulence. Lung cancer surgery has always been an ultramajor undertaking with risks of morbidity for each patient, despite the advances made in minimally invasive techniques.^5^ Experience with conventional neoadjuvant/induction therapy using chemo‐ and/or radiotherapy has shown that any such combined therapy only adds to the potential for complications and compromised surgical outcomes.^6^ With immunotherapy, there is no reason to think the same will not also occur. Early experience using immunotherapy in an induction setting has already suggested that the subsequent surgery could be rendered technically more challenging.^7^

In this context, the clinical recommendations presented by Ni et al. in this issue have arrived at a critical juncture.^8^ These recommendations focus precisely on the potential hazards that perioperative immunotherapy may pose for subsequent lung cancer surgery. The authors explore the evaluation of immunotherapy‐related AEs (irAEs), the impact of irAEs on surgery, and the potential strategies of any irAEs that occur. The details of these recommendations should be read in the study itself. However, one wishes to highlight two reasons why these recommendations are so timely.

First, the authors have presciently anticipated the turbulence ahead. As increasing numbers of lung cancer specialists enthusiastically research and practice neoadjuvant/induction immunotherapy followed by surgery, occurrences of irAEs affecting surgical outcomes will inevitably also increase. As this is still a new field of lung cancer medicine, these intrepid early‐adopters will essentially be sailing into uncharted waters. Having a set of clinical recommendations developed from a thorough review of the literature is a vital asset to help them to navigate a safe course for their patients. The caveat is that any set of guidelines cannot be perfect at this stage. As research in this young field accumulates, there will undoubtedly be changes and additions to these clinical recommendations, but they will be building upon the solid foundation provided by these initial recommendations.

Second, the panel of authors that have crafted these clinical recommendations comprises experts from diverse backgrounds, including pulmonologists, oncologists, thoracic surgeons and other lung cancer‐related specialties. This is highly significant. As the waters of river and well swirl and mix, a proper understanding of the turbulence can only be gained when masters of both river and well work together. The multispecialty collaboration marks this set of clinical recommendations as particularly authoritative and balanced. The interests and concerns of surgeons do not outweigh those of oncologists, and vice‐versa. Instead, the oncologist's experience of the side effects from immunotherapy is married to the surgeon's appreciation of the potential points of weakness where perioperative complications can arise. Although obvious, it is worth stressing that multidisciplinary management does require multidisciplinary guidance to safeguard patients.

It is hoped that the publication of these clinical recommendations by Ni et al. is a good sign of things to come. With the advances being made in immunotherapy and other fields of lung cancer medicine, it appears that the progress will usher in an era of increasing opportunities for greater collaborations between specialities. As waters from more rivers merge with those from more wells, the example set here should be carefully considered. That is, turbulence needs to be anticipated, and it should be addressed by a multidisciplinary effort.
